# Fc-modified exenatide-loaded nanoparticles for oral delivery to improve hypoglycemic effects in mice

**DOI:** 10.1038/s41598-018-19170-y

**Published:** 2018-01-15

**Authors:** Yanan Shi, Xinfeng Sun, Liping Zhang, Kaoxiang Sun, Keke Li, Youxin Li, Qiang Zhang

**Affiliations:** 1 School of pharmacy, Binzhou Medical University, Yantai, 264003 China; 2 School of Pharmacy, Yantai University, Yantai, 264005 China; 3 State Key Laboratory of Long-acting and Targeting Drug Delivery System, Luye Pharmaceutical Co, Ltd, Yantai, 264003 China; 4 Beijing Key Laboratory of Molecular Pharmaceutics and New Drug Delivery Systems, School of Pharmaceutical Sciences, Peking University, Beijing, 100871 China

**Keywords:** Nanoparticles, Peptide delivery

## Abstract

To improve the oral efficiency of exenatide, we prepared polyethylene glycol-poly(lactic-co-glycolic acid) (PEG-PLGA) NPs modified with Fc (NPs-Fc) for exenatide oral delivery. Exenatide was encapsulated into the NPs by the w/o/w emulsion-solvent evaporation method. The particle size of the NPs-Fc was approximately 30 nm larger than that of the unmodified NPs with polydispersity indices in a narrow range (PDIs; PDI < 0.3) as detected by DLS, and the highest encapsulation efficiency of exenatide in the NPs was greater than 80%. Fc-conjugated NPs permeated Caco-2 cells faster and to a greater extent compared to unmodified NPs, as verified by CLSM and flow cytometry. Hypoglycemic effect studies demonstrated that oral administration of exenatide-loaded PEG-PLGA NPs modified by an Fc group extended the hypoglycemic effects compared with s.c. injection of the exenatide solution. Fluorescence-labeled NPs were used to investigate the effects of Fc targeting, and the results demonstrated that the NPs-Fc stayed in the gastrointestinal tract for a longer time in comparison with the unmodified NPs, as shown by the whole-body fluorescence images and fluorescence images of the dissected organs detected by *in vivo* imaging in live mice. Therefore, Fc-targeted nano-delivery systems show great promise for oral peptide/protein drug delivery.

## Introduction

Exenatide, a 39-amino-acid peptide with a molecular weight of 4186 Da and isoelectric point of pH 4.86, is similar to glucagon like peptide-1 (GLP-1) in terms of glucoregulatory actions and is used for type 2 diabetes therapy^1^. The marketed products include Byetta, with subcutaneous (s.c.) administration twice a day. Thus, long-acting formulations are being developed for clinical application. The sustained release microsphere product of exenatide (Bydureon®) was approved by the FDA in 2012^2^. In addition, an implantable osmotic pump for exenatide with 6 to 12 months delivery (ITCA650) has finished phase 3 clinical trials^3,4^; however, patient acceptance of this implantable device is uncertain. Exenatide administration is limited to parenteral routes, resulting in low patient compliance. Presently, one of the major challenges is the development of a patient-friendly delivery of proteins/peptides in the pharmaceutical field.

Oral delivery is a preferred route for patients due to the convenience and strong compliance. However, oral delivery of proteins/peptides is still challenging mainly because of the instability of these molecules in the gastrointestinal tract, their considerably low permeation efficiency through the intestinal epithelium, and their rapid indigestive degradation^5,6^.

The advance of nanotechnology has opened a new era for protein/peptide oral delivery. Nanocarriers exhibit several prominent advantages, such as protection of proteins via encapsulation in the nanocarriers and mitigation of drug modification. Numerous nanocarriers have emerged for protein oral administration, such as chitosan nanoparticles^5^, starch-based nanoparticles^7^, and liposomes^8^. PEG-PLGA nanoparticles have been studied owing to their great potential for performing a longer circulation time. Additionally, the biocompatibility of the delivery system might be improved by the hydrophilic character of PEG^9^. Moreover, this PEG shell is capable of alleviating the aforementioned barriers, as free PEG chains perform as a protein repellent for protecting the nanoparticles from enzymatic attack in the gastrointestinal tract (GIT); on the other hand, the PEG chains can penetrate the GIT’s mucosal layer, which enhance the cellular uptake of the nanocarriers^10^. However, nanocarriers inefficiently transport through the cellular barriers, eg. the intestinal epithelium. It is necessary to apply a new strategy to overcome these barriers.

Some reports have demonstrated that specific cellular uptake and transepithelial transport can be enhanced by conjugating the nanocarriers surface with specific ligands for epithelial receptors or antibodies^11–13^. Wei Shan reported a cell-penetrating peptide that can mediate high epithelial absorption, benefiting insulin-loaded N-(2-hydroxypropyl) methacrylamide copolymer (pHPMA) derivatives for an oral delivery system^14^. CSKSSDYQC (CSK) targeting peptide modifying insulin-loaded trimethyl chitosan chloride nanoparticles enhanced transport by the clathrin- and caveolae-dependent endocytosis pathway after oral delivery^15^. The surface of the *Ulex europaeus* agglutinin-1 (UEA-1) lectin grafting liposomes yielded improvements in nanoparticle transport across the intestinal barrier via targeting α-L-fucose residues expressed on the apical surface of M cells^16^. Vitamin B12 (VB12)-targeted micelles exhibited better *in vitro* uptake and transport in a model of the intestinal cell monolayer compared to untargeted micelles due to the endocytosis of VB12^17^.

The Fc receptor (FcRn) is expressed in the apical region of epithelial cells in the small intestine and throughout the colon both in fetuses and in adults^18^. Studies from W. He *et al*. have indicated that the pathway of Fc to the basolateral surface of the cell is through a complex network of entangled tubular and irregular vesicles^19^. Fc has been used as a targeted ligand for drug oral delivery. Fc-fused follicle-stimulating hormone reached circulation successfully via the route of oral delivery^20^. Eric M. reported that Fc-conjugated nanoparticles could cross the intestinal epithelium and reach the systemic circulation in mice with a higher absorption efficiency compared with untargeted nanoparticles (13.7% vs 1.2%) by oral administration and that the enhancement of nanoparticles was specifically due to the FcRn^21^. The Fc-conjugated nanoparticles can diffuse through the lamina propria and enter the systemic circulation, which might be explained by Fc fragments binding to FcRn at the apical surface of absorptive epithelial cells, inducing receptor-mediated endocytosis^22^. In addition, fluid-phase pinocytosis could aid NPs-Fc absorption. NPs-Fc has high affinity for the FcRn in the acidic endosome compartments, leading to transcytosis and refraining from lysosomal degradation^23^. On the basolateral side, exocytosis can cause exposure of the nanoparticles to a neutral pH environment in the lamina propria, leading to the release of NPs-Fc^24^.

In the present work, we prepared different PEG-PLGA NPs modified with Fc to improve exenatide oral efficacy. The exenatide-loaded PEG-PLGA NPs conjugating with Fc demonstrated better reduction in blood glucose levels and improvement of *in vivo* absorption. In mice, NPs-Fc was imaged crossing the intestinal epithelium and entering into the lamina propria after oral administration. In several organs, NPs-Fc was also detected after oral administration, demonstrating that NPs-Fc could enter into systemic circulation. Exenatide-loaded NPs-Fc was delivered orally and induced a hypoglycemic response. The above results demonstrated that NPs targeted to the FcRn can cross an epithelial barrier and enter *in vivo* circulation, making oral NPs-based delivery systems profitable to deliver drug to the systemic circulation.

## Results

### Characterization of nanoparticles

**Figure 1:**
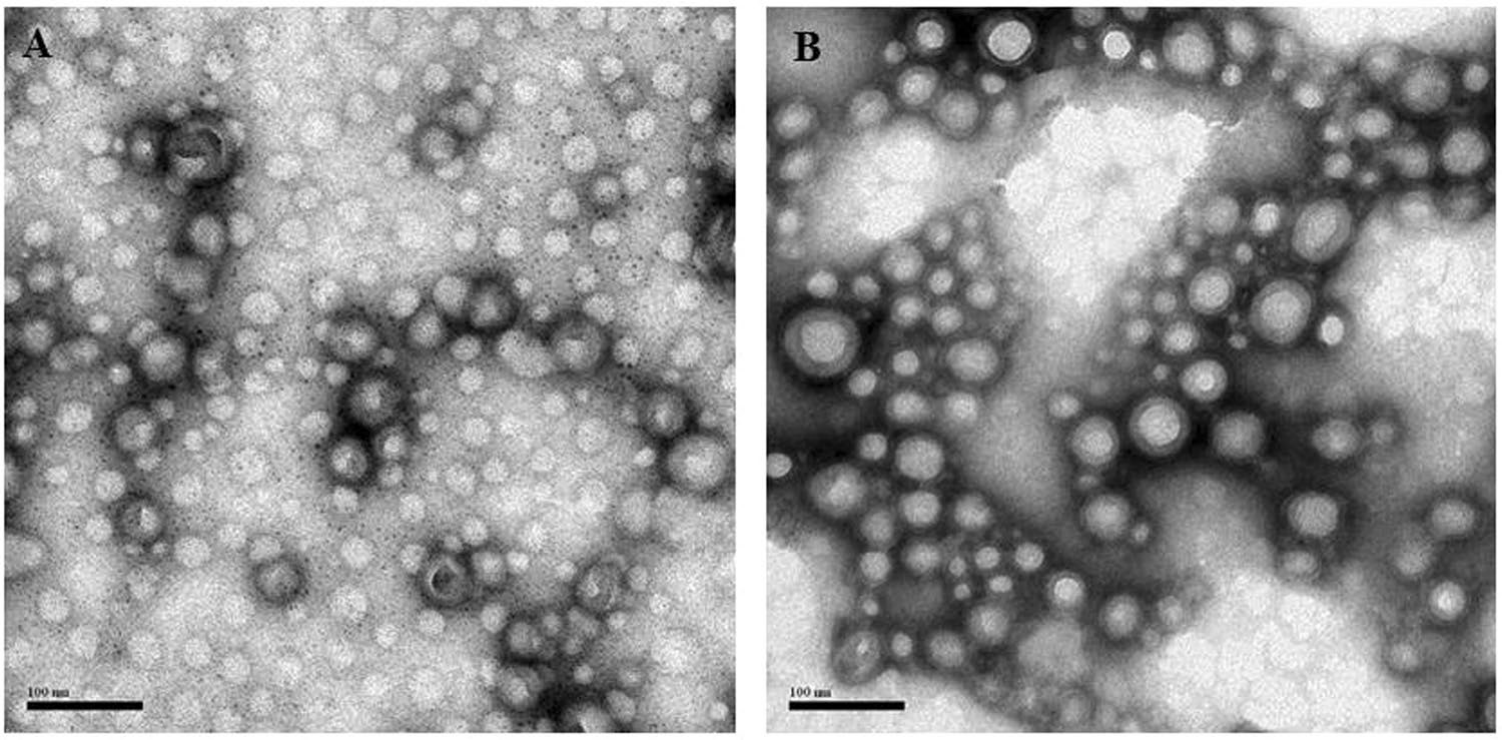
TEM images. (**A**) Exenatide-loaded nanoparticles, (**B**) Exenatide-loaded nanoparticles conjugated with Fc fragments.

The copolymer PEG-PLGA was used to fabricate the exenatide-loaded nanoparticles. Pegylation (PEG) can protect nanoparticles against aggregation and enzymatic degradation, benefiting a longer circulating cycle *in vivo*, which has been used widely to enhance the stability of nanoparticles^25^. In addition, PEG-PLGA forms the hydrophilic corona of the nanoparticles, which can promote mucus penetration. PLGA has been used in many marketed products, and it forms the NPs core owing to its biodegradability and biocompatibility characteristics, as well as long acting release behaviors. PEG-PLGA was used to prepare the nanoparticles by the method of double emulsification. The morphology of PEG-PLGA NPs was detected by TEM. The image (Fig. 1A) showed the dark staining surrounded the bright entities, indicating the presence of spherical particulates, and the NPs were uniform in size.

There was no crucial difference in particle size between the PEG_5000_-PLGA_20000_ NPs and the PEG_5000_-PLGA_28000_ NPs, but the encapsulation efficiency (EE) of the PEG_5000_-PLGA_28000_ NPs was much lower than that of the PEG_5000_-PLGA_20000_ NPs (59.5% vs 83.5%). The effect of the PEG_5000_-PLGA_20000_ concentration (1, 3, and 5 mg/mL) on the characterization of NPs was also studied. The size increased with increased PEG_5000_-PLGA_20000_ concentration; nevertheless, the EE was opposite (Table 1).

**Table 1:** Characteristics of exenitide-loaded NPs.

Batch	Copolymer type	Polymer concentration	Fc conjugation	Particle size (nm)	PDI	Zeta Potential (mV)	EE(%)
1	PEG_5000_-PLGA_20000_	1 mg/mL	No	109 ± 6.7	0.144	−3.56 ± 2.12	83.5 ± 2.8
2	PEG_5000_-PLGA_28000_	1 mg/mL	No	117 ± 7.3	0.079	−4.26 ± 1.04	59.5 ± 3.4
3	PEG_5000_-PLGA_20000_	3 mg/mL	No	127 ± 5.2	0.089	−2.38 ± 1.15	62.9 ± 2.3
4	PEG_5000_-PLGA_20000_	5 mg/mL	No	143 ± 4.9	0.073	−3.25 ± 2.41	52.4 ± 2.9
5	PEG_5000_-PLGA_20000_	1 mg/mL	Yes	140 ± 4.2	0.156	−4.87 ± 1.52	83.5 ± 2.8

Note: The particle size and PDI are detected by DLS method.

**Figure 2:**
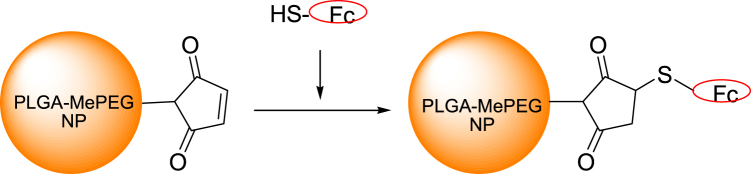
Schematic of the NPs-Fc assembly.

PEG_5000_-PLGA_20000_ with a free terminal maleimide group (PLGA-PEG-MAL) was used to conjugate the Fc portion of the IgG as shown in Fig. 2. Fc was modified by 2-iminothiolane to form thiol groups (Fc-SH). Sephadex G50 gel columns were used to separate the unbound Fc-SH from NPs. Fc fragments conjugated onto the surface of the NPs promoted different characterizations of NPs. On one hand, TEM showed that Fc-conjugated NPs exhibited the core-shell structure (Fig. 1B) while the non-conjugated NPs did not (Fig. 1A). On the other hand, Fc-conjugated NPs had a larger size of 130 ± 5.2 nm, approximately 20 nm larger than non-conjugated NPs (Table 1). However, the zeta potential was almost the same for both groups.

### Cellular uptake: imaging by CLSM

**Figure 3:**
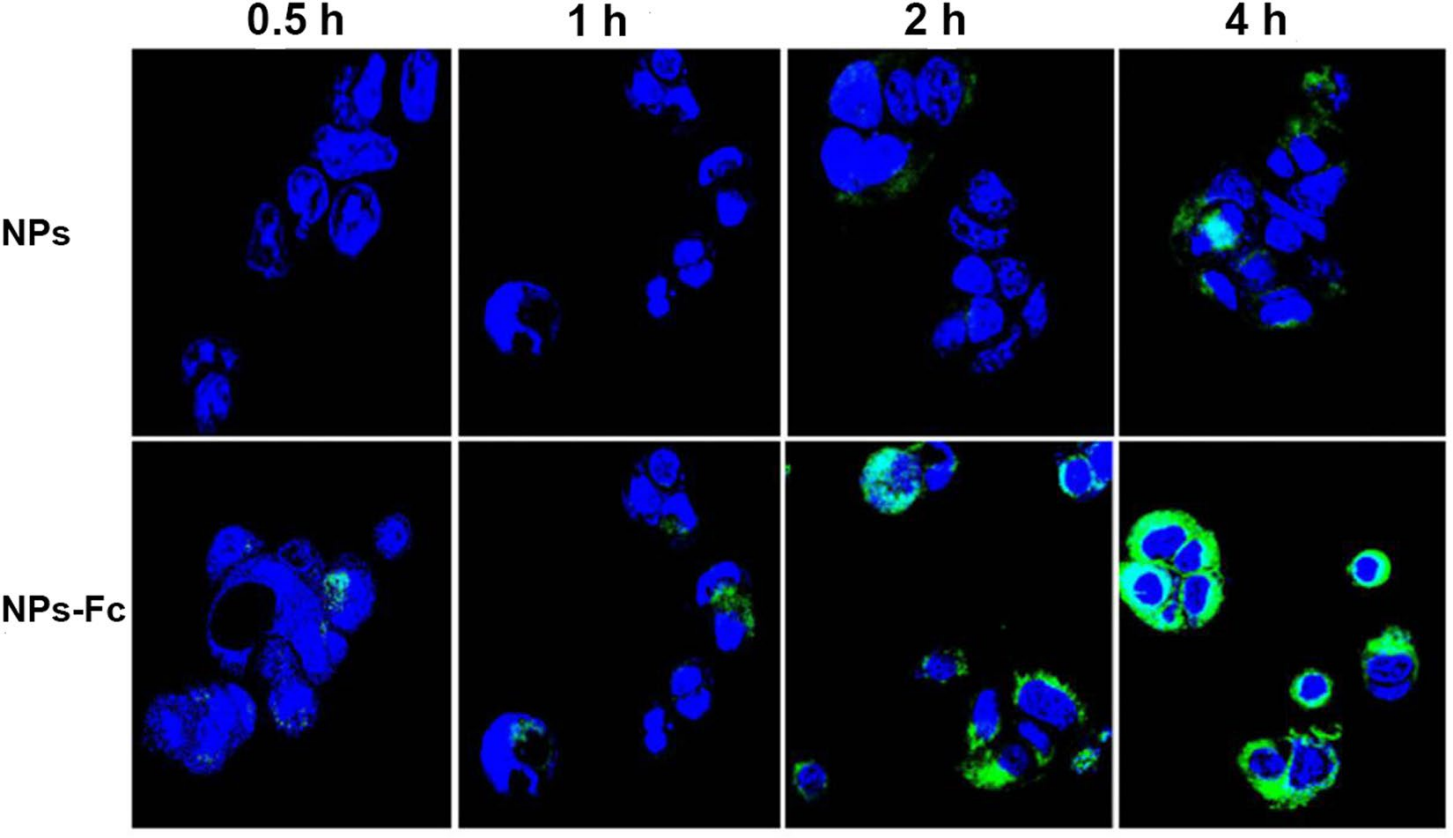
Confocal microscopy images of fluorescently labeled NPs and NPs-Fc incubation with Caco-2 cell for 0.5, 1, 2, and 4 h.

Caco-2 is a cell line of human epithelial colorectal adenocarcinoma that is widely applied for permeability researches in pharmaceutical studies. Therefore, the Caco-2 cell line was used as an epithelial cell monolayer model for drug permeability testing in our research. Due to the endogenous expression of human FcRn and human β_2_-microglobulin in Caco-2 cells, these cells have previously been used for IgG transcytosis studies^26^. The CLSM method was used to detect the corresponding uptake behavior of the NPs and NPs-Fc. The image of a representative Caco-2 cell following incubation with NPs and NPs-Fc is shown in Fig. 3. Green fluorescence (fluorescent NPs) increased with the incubation time, which demonstrated that NPs and NPs-Fc had entered into the Caco-2 cells monolayer. Moreover, the results demonstrated that NPs-Fc was transported into Caco-2 cells within 0.5 h, confirming that the Fc-conjugated NPs could permeate through the Caco-2 cell monolayer more quickly than unconjugated NPs (2 h). After 4 h, the amount of NPs-Fc that permeated into Caco-2 cells was greater than that of unconjugated NPs.

### Cellular uptake: a quantitative analysis

**Figure 4:**
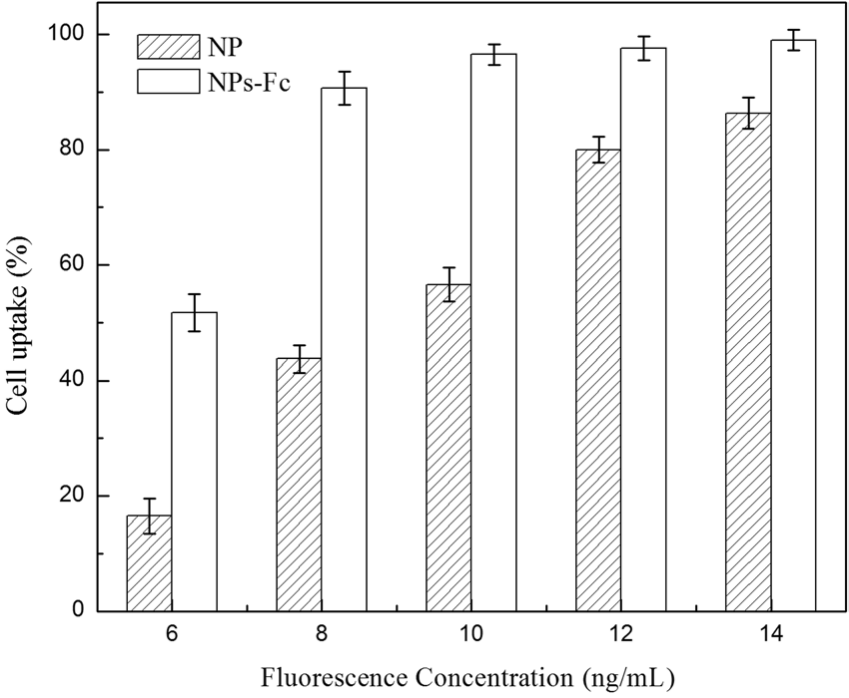
Flow cytometry results of the cellular uptake of fluorescently labeled NPs and NPs-Fc at different fluorescence concentrations.

Cellular uptake of the NPs following an incubation period of 90 min was detected by flow cytometry and is shown in Fig. 4. Unmodified NP internalization increased with the increasing concentration (6–14 ng•mL^−1^). Cellular uptake of Fc-conjugated NPs with a fluorescence concentration of 8 ng•mL^−1^ was almost saturated during the period. This phenomenon demonstrated the Fc-conjugated NPs permeated Caco-2 cells more and faster. Furthermore, internalization of the Fc-conjugated NPs was much more than the unconjugated NPs.

### Hypoglycemic effect studies

**Figure 5:**
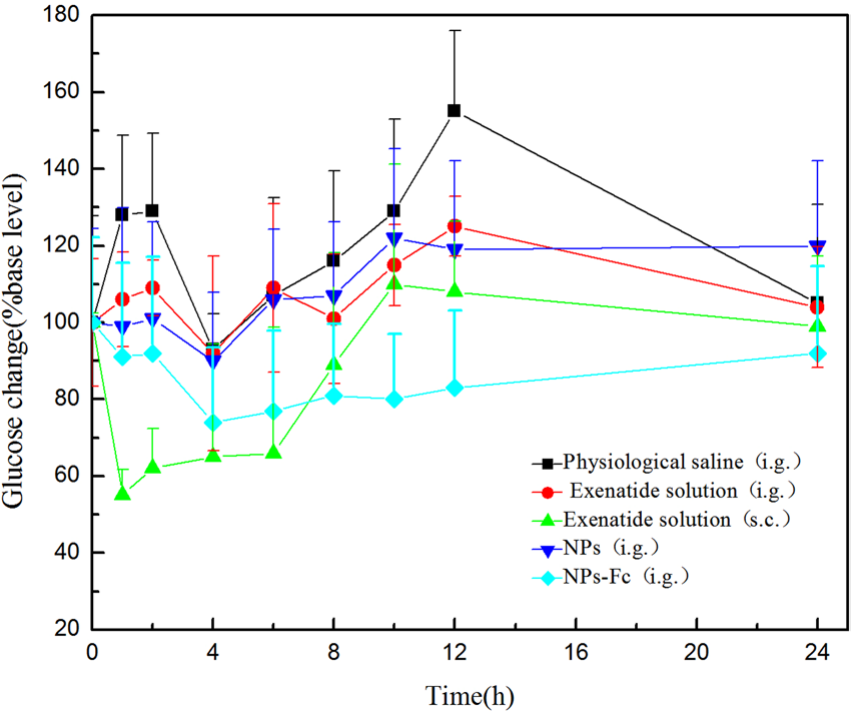
Blood glucose levels in db/db mice following oral administration of the NPs, NPs modified by Fc, exenatide solution and physiological saline, and subcutaneous injection of exenatide solution.

The hypoglycemic effect of exenatide-loaded PEG-PLGA NPs and exenatide-loaded PEG-PLGA NPs modified by Fc was studied in db/db mice after oral delivery. As shown as Fig. 5, the baseline was the average value of blood glucose detected before the treatment for each experimental group, taken as 100%. Both the NPs and NPs-Fc showed significantly high hypoglycemic effects, comparing with the orally administered exenatide solution, at 1 and 2 h after administration. Oral administration of physiological saline made no sense in the blood glucose level but had the opposite effect. The oral exenatide solution group also did not show reduced blood glucose levels, demonstrating poor oral absorption of the free exenatide in the small intestine.

The hypoglycemic effects of the oral administration of PEG-PLGA NPs modified by Fc showed extended time compared with s.c. injection of the exenatide solution. Furthermore, the hypoglycemic effect of the NPs modified by Fc was much more significant than that of the unmodified NPs at every time point.

### *In vivo* image study

In the research, Dir-loaded NPs were used to study the NPs’ distribution. The biodistribution of the orally delivered Dir-loaded NPs in mice was studied using an *in vivo* imaging system.

**Figure 6:**
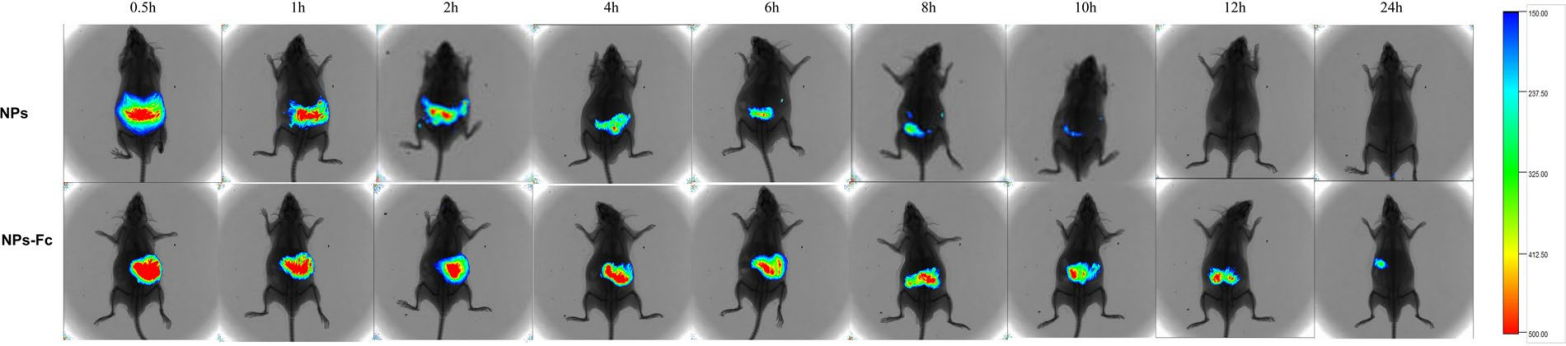
*In vivo* fluorescence images of the whole body post-administration of Dir-loaded NPs and Dir-loaded NPs modified by Fc, respectively, at 0.5, 1, 2, 4, 6, 8, 10, 12 and 24 h.

Figure 6 shows the whole-body fluorescence at 0.5, 1, 2, 4, 6, 8, 10, 12 and 24 h post-oral administration of the Dir-loaded NPs and Dir-loaded NPs modified by Fc. The fluorescence of the group treated with the NPs modified by Fc lasted much longer in the gastrointestinal tract than the unmodified NPs.

**Figure 7:**
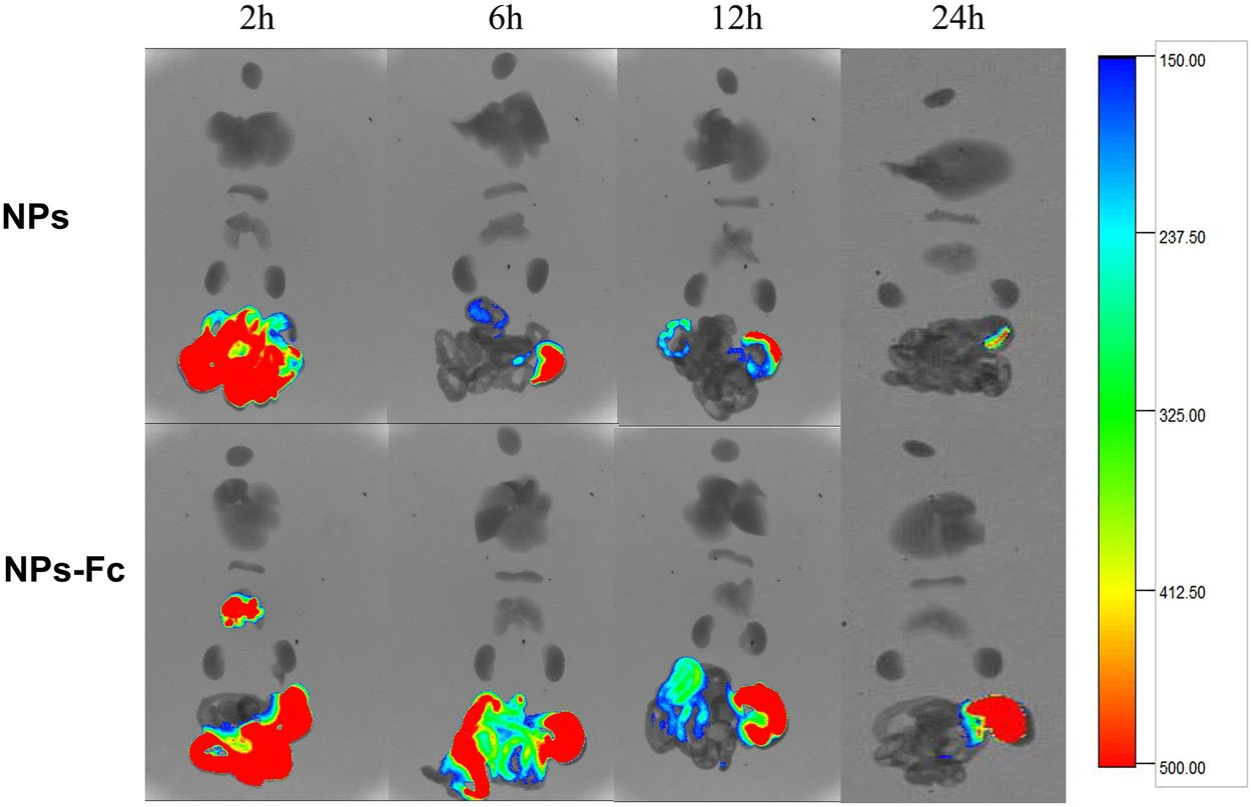
*In vivo* fluorescence images of the anatomized major organs (heart, liver, spleen, lung, kidney and gastrointestinal tract) at 2, 6, 12 and 24 h post-oral administration of Dir-loaded NPs and Dir-loaded NPs modified by Fc, respectively.

Figure 7 shows the fluorescent images of major organs (heart, liver, spleen, lung, kidney and gastrointestinal tract) at the time points (2, 6, 12, and 24 h post-oral administration) of Dir-loaded NPs and Dir-loaded NPs modified by Fc. As for the fluorescent images of excised organs, the higher fluorescence accumulation in the gastrointestinal tract was further confirmed for the group treated orally with the NPs modified by Fc after 2 h. Moreover, the Fc-modified NPs remained in the gastrointestinal tract for a longer time compared with the unmodified NPs. Fluorescence was still observed for the group orally treated with NPs modified by Fc after 24 h.

## Discussion

The current treatment for diabetes is mainly dependent on frequent injections for chronic disease treatment, leading to poor patient compliance. The major problems associated with the outcome is forgetting a dose due to multiple doses needed every day and needle phobia^2^. Considering the requirement of chronic treatment for diabetes, the development of a more patient-compliant route of delivery is urgently needed. Oral delivery has always been the preferred route of administration^27^. However, oral delivery of protein and peptide drugs faces significant technical hurdles due to the rapid degradation of these drugs by gastric enzymes, the acidic environment, and poor permeation across the intestinal epithelium^2^.

Here, we aimed to improve exenatide oral absorption by conjugating the specific targeting ligand Fc to the nanoparticle surface. The efficacy of oral Fc-modified exenatide-loaded PEG-PLGA NPs was studied to provide a non-invasive route for diabetes treatment as an alternative to frequent injections. The Fc-modified exenatide-loaded PEG-PLGA NPs were able to target the FcRn for epithelial transport and enhanced intestinal absorption due to the FcRn-mediated effect of transcytosis of IgG across several epithelial and endothelial barriers^5^. Qiao reported that targeting FcRn NPs retained the integrity of the epithelia barrier in the transcytosis process with regard for the potential safety issues associated with the permeability of the intestine^28^. Our research demonstrated that the IgG Fc-modified NPs crossed the intestinal epithelium and reached systemic circulation. The targeting NPs were capable of improving exenatide oral bioavailability and imparting a significant hypoglycemic effect.

PEG-PLGA, the carrier material of NPs, is crucial for drug encapsulation. It was found that the EE of the PEG_5000_-PLGA_28000_ NPs was much lower than that of the PEG_5000_-PLGA_20000_ NPs, which might be because the hydrophobic attributes of an amphiphilic block copolymer carrier material increase with an increase in the molecular weight of PLGA, leading to a lower capacity of NPs to encapsulate hydrophilic exenatide. The similar phenomenon of lower encapsulation efficiency appeared at a higher PEG-PLGA concentration, which might also be explained by an enhancement of hydrophobic attributes. Furthermore, there was an increase in particle size with the increase in hydrophobic PLGA molecular weight or PEG-PLGA copolymer concentration, which can be explained by an enlarged hydrophobic radius with an increase in the hydrophobic nature of the carrier material.

Both TEM and DLS results showed that Fc was successfully conjugated onto the surface of NPs. As shown as Fig. 2B, the TEM image of NPs-Fc exhibited the core-shell structure, which was not present in non-conjugated NPs (Fig. 2A). The NPs-Fc promoted a diameter approximately 30 nm larger than non-conjugated NPs as detected by DLS, due to the Fc fragment exhibiting a hydrodynamic diameter. A similar conclusion was reported by a previous study^29^. Generally, TEM showed much smaller particle size than that determined by DLS, resulting from the influences of the dehydration and collapse of the hydrophilic PEG corona of the nanoparticles^30^.

Particle size is an important factor to the absorption, distribution, and *in vivo* behaviors of nanoparticles. In our research, PEG-PLGA NPs and Fc-modified PEG-PLGA NPs with particle sizes of 109 nm and 130 nm, respectively, have higher cellular uptake efficiency, consistent with Bakhru *et al*.^31^ and Panyam and Labhasetwar^32^, whose studies reported that the cellular uptake of nanoparticles with a particle size of 100 nm posed much higher cellular uptake efficiency than larger particles (microparticles) *in vitro* ^31^ and *in vivo* ^32^. This phenomenon can be explained by the mechanisms of translocation of NPs in the intestinal epithelium involving better absorption of NPs in Peyer’s patches^33^.

It was noticed that the Fc-modified PEG-PLGA NPs entered Caco-2 cells more quickly and to a greater extent than the unmodified NPs, which could be because the FcRn receptor markedly enhanced the transepithelial transport of the Fc-modified NPs. The FcRn receptor is expressed throughout the intestine, performing a remarkable increase in the available surface area for absorption of Fc-modified NPs, which is much better than other dose forms that target only a specific portion of the intestine, such as the Peyer’s patches^34^. Though Fc-modified PEG-PLGA NPs exhibited a better Caco-2 cells uptake based on our data, which cell uptake mechanism is not clear, therefore, we would do more research about the cell uptake mechanism, e.g., Transport through cell monolayers study.

The mechanism of action of exenatide is capable of stimulating β-cells to release insulin under hyperglycemic conditions, whereas without stimulation of insulin secretion, the glucose concentration is in the normal range^35^. Therefore, exenatide cannot promote severe hypoglycemic events^18,36^. The efficacy of the PEG-PLGA NPs and the Fc-modified NPs following oral administration was investigated in db/db mice. The hypoglycemic effect study demonstrated that all the oral NPs had a better hypoglycemic response compared to the oral exenatide solution. The FcRn-targeting NPs also reduced the glucose level more significantly than the unmodified NPs. In addition, the hypoglycemic response of the FcRn-targeting NPs lasted for 12 h, which was longer than in the exenatide s.c. administration group (8 h). It is noteworthy that the FcRn-targeting NPs maintained a lower glucose level that was relatively steady compared with the exenatide s.c. administration. After 24 h, the glucose level remained lower than that before dosing of the targeting NPs, which was similar with previous report and might be due to a hypoglycemic agent^15^.

Figure 6 shows the whole-body fluorescence *in vivo* image and demonstrates that the Fc-modified NPs were retained in the gastrointestinal tract for a longer time (24 h) than the unmodified NPs (10 h). Moreover, as shown in Fig. 7, the dissected major organs image showed that more fluorescence remained in the gastrointestinal tract and remained longer for the Fc-modified NPs group compared with the unmodified NPs group. In addition, NPs in the modified group entered the lung and kidney portions. All these phenomena demonstrated that the Fc-modified NPs exhibited better gastrointestinal targeting attributes and benefitted the drug absorption *in vivo*. Furthermore, it was reported previously that rodents could remarkably down-regulate FcRn expression in the intestine after weaning^18^, while humans continue to express FcRn into adulthood. According to this theory, the transport of Fc-modified NPs in humans could potentially be even more efficient in comparison with transepithelial transport in mice. Therefore, the Fc-targeting drug delivery system is promising for orally delivered treatments in humans.

Overall, the *in vivo* hypoglycemic effect and *in vivo* imaging results were in accordance with the *in vitro* cellular level studies. Fc-modified NPs are promising for peptide or protein oral delivery. However, much additional research is needed to demonstrate the oral absorption of exenatide, e.g., pharmacokinetic study or the mechanism of transport through Caco-2 cell research.

In the present work, a novel formulation of Fc conjugated to exenatide-loaded PEG-PLGA NPs was developed and characterized. Exenatide was efficiently encapsulated by PEG-PLGA nanoparticles or Fc-modified PEG-PLGA nanoparticles. The Fc-modified NPs were retained in the gastrointestinal tract for a longer time and exhibited easier cellular uptake to promote a preferable hypoglycemic effect in db/db mice. All the findings from the *in vitro* and *in vivo* studies demonstrate that an Fc-targeting nano-delivery system shows great promise for oral peptide or protein drug delivery.

## Methods

### Material

Exenatide was purchased from Gill Biochemistry Co., Ltd. (Shanghai, China). PEG-PLGA-maleimide (mal-PEG-PLGA; 5,000–20,000 Da, 50:50 LA:GA, w/w) and methoxy-PEG-PLGA (mPEG-PLGA; 5,000–20,000/25000/28000 Da, 50:50 LA:GA, w/w) were purchased from Polyscitech (West Lafayette, IN, USA). Human polyclonal IgG Fc was purchased from Laibao Experimental Equipment Co., Ltd. (Zhengzhou, China). An exenatide enzyme-linked immunosorbent assay (ELISA) kit was bought from Phoenix Pharmaceutics, Inc., USA. Accu-Chek Integra test strips were obtained from Roche Diagnostics GmbH (Shanghai, China). All other chemicals used were of analytical grade.

### Preparation of PEG-PLGA nanoparticles

A double emulsion method was used to prepare Fc-modified nanoparticles (NPs) as previously reported^21^. Briefly, an appropriate amount of mPEG-PLGA and PLGA-PEG-mal in a ratio of 9:1 was dissolved in 1 mL acetone and dichloromethane to form the oil phase, and 400 μL exenatide aqueous solution (2.5 mg/mL) or distilled water was added to the oil phase, and emulsified by probe sonication (300 W, JY92-II ultrasonic processor, Ningbo Scientz Biotechnology Co. Ltd., China) in an ice bath to form the first emulsion. Next, the emulsified mixture was added to 2 mL 3% (w/v) PVA aqueous solution, followed by ultrasonication (300 W, JY92-II ultrasonic processor, Ningbo Scientz Biotechnology Co. Ltd., China) in an ice bath to form the double emulsion. Afterwards, the double emulsion was added to 20 mL 0.5% (w/v) PVA aqueous solution and stirred for 3 h at room temperature to evaporate and remove the organic solvents (DCM and acetone). Then, the nanoparticles were obtained.

### Morphology of the nanoparticles

The appearance of the nanoparticles was observed by a transmission electron microscope (TEM) (JEOL JEM-1400, Japan Field Co., Ltd.). The NP solution was dropped in a carbon-coated copper grid and negatively stained with a 2% solution of phosphotungstic acid followed by air drying. The acceleration voltage was set at 80 kV.

### Particle size and zeta potential

The obtained exenatide-loaded PEG-PLGA nanoparticles were characterized for particle size, PDI and zeta potential by a DLS instrument (NicompTM380/ZLS PSS, USA) operating at 633 nm and room temperature. The light scattering angle was set at 90°.

### Encapsulation efficiency

The nanoparticles were ultrafiltered using Vivaspin ultrafiltration spin columns (MW cutoff = 100 kDa) and centrifuged at a rotational speed of 3,500 rpm for 30 min at 4 °C (Centrifuge, Sorvall Biofuge Primo R) to remove free exenatide. The NPs were washed with water twice. Exenatide was extracted from nanoparticles for the determination of the exenatide by ultrasonication with methanol. Exenatide content was determined using an Agilent 1260 HPLC system equipped with an chromatographic column (Ultimate® LP-C18, 4.6 mm × 250 mm, 5 μm pore size, Agilent) and a gradient elution of (A) acetonitrile containing 5% phosphoric acid and (B) water containing 5% phosphoric acid as the mobile phase. The method utilized was 0 min (30% A) → 10 min (44% A) → 10.1 min (30% A) → 15 min (30% A). The flow rate was set at 1 mL/min, and the elution temperature was 40 °C. The retention time of exenatide was 9.5 min, and the total run time for the HPLC analysis was 15.0 min.

The encapsulation efficiency (EE) was obtained by the formula as follows:

$${\rm{EE}}\,( \% )={\rm{exenatide}}\,{\rm{encapsulated}}\,{\rm{in}}\,{\rm{the}}\,{\rm{NPs}}/{\rm{total}}\,{\rm{exenatide}}\times \mathrm{100} \% $$

### Synthesis and characterization of Fc-modified PEG-PLGA nanoparticles

Human polyclonal IgG Fc (1.5 mg) in PBS was reacted with 41.85 μL of 2-iminothiolane (1 mg/mL; Traut’s Reagent) for 1 h. The modified Fc was added to the NPs and mixed for 1 h at 4 °C to allow conjugation^21^. The NPs-Fc were washed with PBS using Vivaspin ultrafiltration spin columns (MW cutoff = 100 kDa). Free IgG Fc was measured with a protein bicinchoninic acid (BCA) assay from Beyotime Biotechnology. Particle size, PDI and zeta potential were measured using the same method as above.

### Coumarin-6-loaded NPs

For the fluorescence imaging cell study, the fluorescent probe Coumarin-6 with a high laser conversion rate was loaded into the NPs. The method was similar to that used for exenatide. The NPs were ultrafiltered using Vivaspin ultrafiltration spin columns (MW cutoff = 100 kDa) and centrifuged to remove free Coumarin-6.

### Dir-loaded NPs

The near-infrared fluorescent probe Dir was encapsulated into the NPs for *in vivo* fluorescence imaging. The preparation method was corresponding to that used for exenatide. The NPs were ultrafiltered using Vivaspin ultrafiltration spin columns (MW cutoff = 100 kDa) and centrifuged to remove free Dir.

### Cell culture

Human colon carcinoma Caco-2 cells were purchased from the Chinese Academy of Medical Sciences (Shanghai, China). These cells were grown in culture dishes using DMEM supplemented with 15% fetal bovine serum and 1% non-essential amino acids. Both cell types were placed in a cell culture incubator at 37 °C, with 95% relative humidity and 5% CO_2_. After proliferation for 4 days, cells were harvested using trypsin (0.25%) containing ethylenediamine tetra-acetic acid (EDTA, 0.02%). For the cellular uptake study, Caco-2 cells were seeded into 96-well or 6-well plates.

### Intracellular uptake studies

Caco-2 cells were quantitatively and qualitatively studied using flow cytometry (FACS) and confocal laser scanning microscopy (CLSM), respectively.

### Confocal laser scanning microscope

To investigate the cellular interaction with NPs, the fluorescent probe coumarin-6 was encapsulated to PEG-PLGA NPs and Fc-PEG-PLGA NPs. The cellular uptake of NPs was detected by CLSM (FluoView FV1000, Olympus, Japan). Caco-2 cells were seeded into 24-well plates at a density of 1 × 10^5^ cells/mL. The NPs were co-incubated with the cells for 0.5, 1, 2, 3, and 4 h at a dose of 200 ng/mL (coumarin-6). The excitation wavelength was 466 nm, and the emission wavelength was 504 nm^26^.

### Flow cytometry

For flow cytometry, Caco-2 cells were seeded in 24-well cell culture plates at a density of 5 × 10^5^ cells per well and allowed to adhere for 48 h until confluency. Then, fluorescence microscopy was performed to observe the cellular uptake. Coumarin-6-loaded PEG-PLGA NPs and coumarin-6-loaded Fc-conjugated PEG-PLGA NPs (coumarin-6 concentrations: 6, 8, 10, 12, and 14 ng•mL^−1^) were co-incubated with the cells for 90 min, and then, the cells were washed three times with PBS and detached from the plates by trypsinization. The cells were then centrifuged at 1,500 rpm for 5 min (Sorvall Biofuge Primo R; Thermo Fisher Scientific, Waltham, MA, USA). Afterwards, the supernatant was discarded, and the cells were resuspended in PBS. Fluorescence was measured using a BD FACSAria^TM^ flow cytometer^37^.

### Hypoglycemic effect studies

In this study, db/db mice were used as a model of type II diabetes. All experiments were performed according to the Institutional Animal Care and National Institutes of Health Guidelines for the Care and Use of Laboratory Animals (USA), and the protocol was approved by the Committee on the Ethics of Animal Experiments of Binzhou Medical University (Permit No. SCXK20140005). Glucose concentrations were measured using a glucose meter (ACCU-CHEK® Integra, Germany).

Diabetic mice, eight per group, were treated with the following: oral administration of exenatide-loaded PEG-PLGA NPs (100.0 μg/kg exenatide), exenatide-loaded Fc-PEG-PLGA NPs (100.0 μg/kg exenatide), exenatide solution (100 μg/kg exenatide) or a s.c. injection of exenatide solution (10 μg/kg). Saline was injected (s.c.) to a control group. Blood samples were collected from the tail veins at different time intervals (0, 1, 2, 3, 4, 6, 8, 10, 12 and 24 h), and the blood glucose concentration was measured with a glucose meter.

### *In vivo* imaging study

Dir, a hydrophobic fluorescence label, was encapsulated into PEG-PLGA NPs and Fc-PEG-PLGA NPs. The NPs were administered orally to mice at a Dir dose of 0.25 mg/kg to investigate the Fc targeting effects using *in vivo* imaging in live mice. Living mice were monitored at 0.5, 1, 2, 4, 6, 8, 10, 12 and 24 h after administration and organ images were obtained at the 2, 6, 12, and 24 h time points using the *In-Vivo* Imaging System FX Pro (Carestream, NY, USA) with an excitation wavelength of 720 nm and an emission wavelength of 790 nm. The images were analyzed using Carestream Image Suite Software^38^.

### Data availability

No datasets were generated or analyzed during the current study
