# Author Correction: Fc-modified exenatide-loaded nanoparticles for oral delivery to improve hypoglycemic effects in mice

**DOI:** 10.1038/s41598-023-39548-x

**Published:** 2023-08-02

**Authors:** Yanan Shi, Xinfeng Sun, Liping Zhang, Kaoxiang Sun, Keke Li, Youxin Li, Qiang Zhang

**Affiliations:** 1 School of Pharmacy, Binzhou Medical University, Yantai, 264003 China; 2 School of Pharmacy, Yantai University, Yantai, 264005 China; 3 State Key Laboratory of Long-Acting and Targeting Drug Delivery System, Luye Pharmaceutical Co, Ltd, Yantai, 264003 China; 4 Beijing Key Laboratory of Molecular Pharmaceutics and New Drug Delivery Systems, School of Pharmaceutical Sciences, Peking University, Beijing, 100871 China

Correction to:* Scientific Reports* 10.1038/s41598-018-19170-y, published online 15 January 2018

This article contains errors.

As a result of an error during figure assembly in Figure 3, the image for NPs at 1 h shows blue channel image for NPs-Fc at 1 h. The correct Figure 3 is shown below.

**Figure 3:**
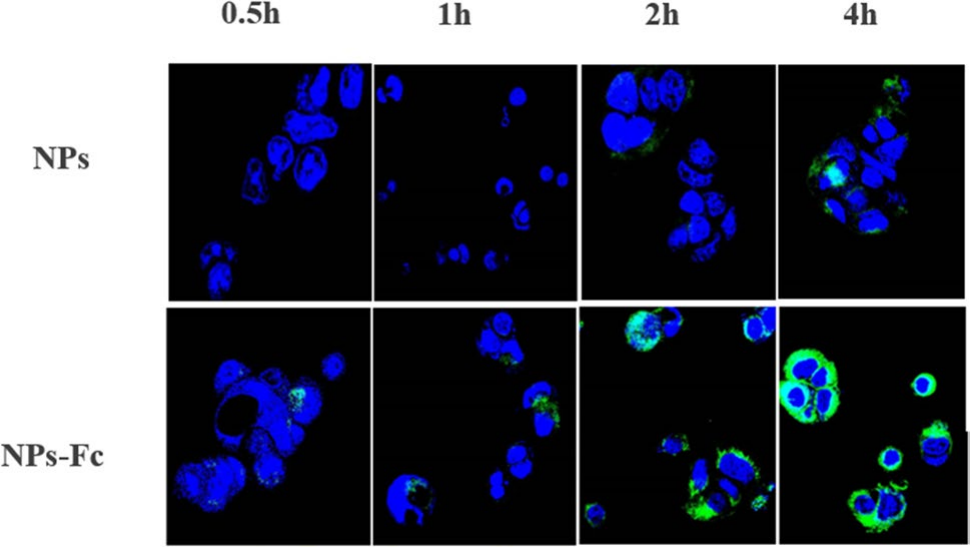
Confocal microscopy images of fluorescently labeled NPs and NPs-Fc incubation with Caco-2 cell for 0.5, 1, 2, and 4 h.

